# Locally kernel weighted maximum likelihood estimator for local linear multi-predictor poisson regression

**DOI:** 10.1016/j.mex.2025.103258

**Published:** 2025-03-09

**Authors:** Darnah, Memi Nor Hayati, Sri Wahyuningsih, Iriyani Kamaruddin, Suyitno, Andrea Tri Rian Dani, Rito Goejantoro, Desi Yuniarti, Fidia Deny Tisna Amijaya, Ika Purnamasari, Meiliyani Siringoringo, Surya Prangga, Ratna Kusuma, Rahmawati Munir

**Affiliations:** a Department of Mathematics, Faculty of Mathematics and Natural Sciences, Mulawarman University, Indonesia; b Department of Public Health, Faculty of Public Health, Mulawarman University, Samarinda, Indonesia; c Department of Biology, Faculty of Mathematics and Natural Sciences, Mulawarman University, Samarinda, Indonesia; d Department of Physics, Faculty of Mathematics and Natural Sciences, Mulawarman University, Samarinda, Indonesia

**Keywords:** Local linear, Poisson regression, Kernel, Newton-Raphson, MLCV, Stunting, Locally Kernel Weighted Maximum Likelihood Estimator for Local Linear Multi-predictor Poisson Regression

## Abstract

We introduce a new multi-predictor regression model based on the Poisson distribution using a local linear approach called the local linear multi-predictor Poisson regression. The optimal bandwidth in this study was selected based on the maximum likelihood cross-validation (MLCV) value. The locally kernel-weighted maximum likelihood estimator is used to estimate the regression curve at a given point. Parameter estimation was performed using the Newton-Raphson iteration method. The superior points in this research are:

• A new model in regression to model multi-predictor case Poisson regression problems using a local liner approach
• Optimal bandwidth selection using MCLV
• Application of multi predictor case Poisson regression problems using a local liner approach to health data; namely the stunting case in East Kalimantan

## Graphical abstract

**Figure:**
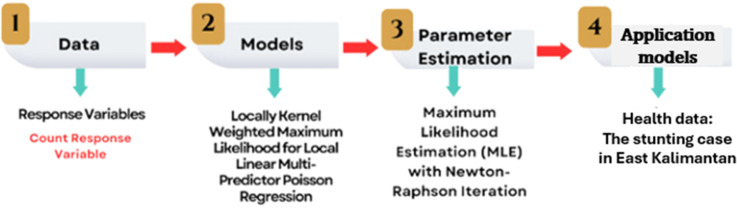

Specifications table

**Table:** 

Subject area:	Mathematics and Statistics
More specific subject area:	*Statistical Modeling; Nonparametric Regression; Poisson Regression; Regression Analysis; Multi-predictor*
Name of your method:	*Locally Kernel Weighted Maximum Likelihood Estimator for Local Linear Multi-predictor Poisson Regression*
Name and reference of original method:	*Darnah,* M*. I. Utoyo, and* N*. Chamidah, “Modeling of Maternal Mortality and Infant Mortality Cases in East Kalimantan using Poisson Regression Approach Based on Local Linear Estimator,” in IOP Conference Series: Earth and Environmental Science, Institute of Physics Publishing, Apr. 2019.* doi:10.1088/1755-1315/243/1/012*,023.* *D. Darnah,* M*. Imam Utoyo, and* N*. Chamidah, “Estimation of the Bi-response Poisson Regression Model Based on Local Linear Approach,” International Journal of Academic and Applied Research,* vol*. 3, no. 5, pp. 14–18, 2019, [Online]. Available:*http://www.ijeais.org/ijaar *Darnah, I. Utoyo, and* N*. Chamidah, “Bi-response Poisson regression model for modeling effect of early marriage on maternal and infant mortality,” AIP Conf Proc,* vol*. 2554, no. 1, p. 030,005, Jan. 2023, doi:*10.1063/5.0104404*.*
Resource availability:	*None*

## Background

Regression analysis, a statistical method, determines the functional relationship between a response and predictor variables [[1], [2]-3]. In regression models, the response variable can be either a continuous or a count random variable, such as in Poisson regression, Generalized Poisson regression, and Negative Binomial regression [[4], [5], [6], [7]-8]. Poisson regression, a popular model for counting response variables, is particularly useful for studying the occurrence rate per unit of time conditional on some predictors [5,9]. It's important to note that the response variable in Poisson regression must satisfy the equidispersion assumption, where the sample mean equals the variance [7,10]. This is a key property that you should be aware of, as it significantly influences the interpretation of the results [10,11].

The Poisson regression function can be estimated using parametric and nonparametric regression [12]. Parametric regression is used when the form of the regression function is known, and the parameters are estimated [13]. Research on Poisson regression models with parametric regression approaches has been extensively conducted [9]. In practice, the relationship between the predictor and response variables often does not follow a specific pattern but tends to be irregular. Parametric regression in such conditions may lead to inaccurate conclusions, so nonparametric regression is used to address this issue [13]. Nonparametric regression offers high flexibility since the regression function's form is not specified but is assumed to be smooth, and it is estimated using specific smoothing methods based on the data pattern [[14], [15]-16].

Several estimators are frequently used in nonparametric regression approaches, for example: local linear [[17], [18], [19], [20]], local polynomial [[21], [22], [23]-24], kernel [[25], [26]-27], spline [[28], [29]-30], penalized spline [[31], [32]-33], and truncated spline [16,34]. According to Darnah [35], the local linear estimator is commonly employed for estimating regression functions due to its simplicity, ease of implementation, suitability for fixed and random designs, and ability to perform well near boundaries. Baillo and Grane [36] compared local linear estimators to kernel estimators and determined that the local linear estimator is superior to the kernel estimator. Local linear approximation for single predictor Poisson regression has been developed by Darnah [4,12,35]. However, previous research was limited to a single predictor; therefore, this study proposes a multi-predictor regression model based on the Poisson distribution using a local linear approach called the local linear multi-predictor Poisson regression.

The discussion that follows in this paper is divided into several primary topics. The Poisson regression model is explored through a local linear approach for the predictor variables, following the definition of the regression model. We present the primary findings, encompassing the definition of multi-predictor Poisson Regression and the local kernel-weighted maximum likelihood estimator method. The Newton-Raphson iteration method is followed by a detailed discussion of the maximum parameter estimation and the optimal bandwidth selection using maximum likelihood cross-validation. The local linear multi-predictor Poisson regression we apply the regression model that we propose to health data.

## Method details

### Poisson regression model using local linear approach

The Poisson regression model is a generalized linear model where the response variable follows a Poisson distribution, which is part of the exponential family of distributions. In a generalized linear model, there are three key components: the random component, the systematic component, and the link function [36]. Let $Y_{i}\;\;\left(i=1,\;2,\;\cdots ,\;n\right)$ be the random variable for count data and it follows a Poisson distribution, the probability density function (PDF) is

(1) $$P\left(Y_{i}=y_{i}|x_{i}\right)=f\left(y_{i}|x_{i}\right)=\frac{\theta {\left(x_{i}\right)}^{y_{i}}\exp\left(-\theta (x_{i})\right)}{y_{i}!},\;\;y_{i}=0,\;1,\;2,\;\ldots$$

with mean and variance,

(2) $$E\left(Y_{i}|x_{i}\right)=Var\left(Y_{i}\right)=\theta \left(x_{i}\right)$$

Eq. (1) can be rewritten in the form of the probability density function (PDF) of the exponential family of distributions as

(3) $$P\left(Y_{i}=y_{i}|x_{i}\right)=f\left(y_{i}|x_{i}\right)=\exp[y_{i}\log\left(\theta \left(x_{i}\right)\right)-\theta \left(x_{i}\right)-\log\left(y_{i}!\right)$$

where $Y_{i}$ is a random component, $E\left(Y_{i}\right)=\theta \left(x_{i}\right)$ and $g\left(\theta \left(x_{i}\right)\right)=\log\left(\theta \left(x_{i}\right)\right)$ is canonical link function. The link function establishes a connection between the random component and the systematic component, where $\tau ={\underset{˜}{x}}^{T}\underset{˜}{\beta }$, creating a relationship between the response variable Y and the p predictors X. This relationship is referred to as the Poisson regression model, which is expressed as

(4) $$\theta \left(x_{i}\right)=\exp\left(m\left(x_{i}\right)\right)$$

with $m(x_{i})$ is regression function. In a parametric regression approach with a single predictor variable, $m\left(x_{i}\right)={\underset{˜}{x}}^{T}\underset{˜}{\beta }$ where ${\underset{˜}{x}}^{T}=\left[\begin{array}{cc} 1 & x_{i} \end{array}\right]$ a vector of predictor variable and $\underset{˜}{\beta }={\left[\begin{array}{cc} \beta_{0} & \beta_{1} \end{array}\right]}^{T}$ a vector of regression parameters. The estimates of the parameters $\underset{˜}{\beta }$ can be obtained using the maximum likelihood method [37].

If regression function, $m(x_{i})$in Eq. (4) is an unknown function and we interest to be estimated through nonparametric regression, namely polynomial local smoothing. Considering Taylor expansion as an approximation to$m(x_{i})$, where $x_{i}$ is in a neighborhood of $x_{0}$

(5) $$m\left(x_{i}\right)\approx \sum_{j=0}^{p}\frac{m^{\left(j\right)}\left(x_{0}\right)}{j!}{\left(x_{i}-x_{0}\right)}^{j}\equiv \sum_{j=0}^{p}\beta_{j}\left(x_{0}\right){\left(x_{i}-x_{0}\right)}^{j};x_{i}\in \left(x_{0}-h,x_{0}+h\right)$$

Eq. (5) can be expressed in vector form, which is

(6) $$m\left(x_{i}\right)={\underset{˜}{x}}_{i}^{T*}{\underset{˜}{\beta }}^{*}$$

If p=1 in Eq. (5) then we have local linear smoothing, Eq. (6) can be expressed

$${\underset{˜}{x}}_{i}^{T*}=\left[\begin{array}{cc} 1 & \left(x_{i}-x_{0}\right) \end{array}\right]\;{\underset{˜}{\beta }}^{*}={\left[\begin{array}{cc} \beta_{0}\left(x_{0}\right) & \beta_{1}\left(x_{0}\right) \end{array}\right]}^{T}$$

The locally Kernel weighted maximum likelihood estimator method is used to estimate the regression curve at point $x_{0}$ in nonparametric regression by first estimating its parameters. Based on Eq. (2), we obtain the log local likelihood function [37,38]

(7) $$\begin{array}{ccc} \ell (\underset{˜}{\beta },x_{0}) & = & \ln\left({\prod_{i=1}^{n}f(y_{i}|x_{i})}^{K_{h}\left(x_{i}-x_{0}\right)}\right)\\ & = & \sum_{i=1}^{n}K_{h}\left(x_{i}-x_{0}\right)\left\{\left[\beta_{0}\left(x_{0}\right)+\beta_{1}\left(x_{0}\right)\left(x_{i}-x_{0}\right)\right]y_{i}-\exp\left[\beta_{0}\left(x_{0}\right)+\beta_{1}\left(x_{0}\right)\left(x_{i}-x_{0}\right)\right]-\ln\left(y_{i}!\right)\right\} \end{array}$$

where K is a symmetric probability density function to be used as a kernel and h > 0 its scaling parameter, or bandwidth [39]. The bandwidth controls smoothness of the fit. If h is too small, the fit becomes too noisy, and the variance increases. Conversely, if h is too large, the fit becomes over-smoothed and important feature may be distorted or lost completely. That is, the fit will have large bias. The bandwidth must be chosen to compromise this bias-variance trade-off [40]. The Cross Validation method is a commonly used technique to determine the optimal bandwidth by minimizing the cross validation (CV) value. In a regression model where the regression curve estimator is constructed using the maximum likelihood method, the optimal bandwidth selection is based on the highest value of the maximum likelihood cross validation (MLCV) [41].

(9) $$MLCV\left(h\right)=\sum_{i=1}^{n}\ln f{\left(y_{i},{\hat{m}}_{-i}\left(x_{i}\right)\right)}^{2};i=1,2,\cdots ,n$$

where ${\hat{m}}_{-i}\left(x_{i}\right)$ is the estimated function at the point $x_{i}$ without including the i^th^ data.

## Method validation

### Model multi-predictor case Poisson regression problems using a local liner approach

Let $y_{i}$ is the count response that follows the Poisson distribution and the predictor variable x as many as p or multi-predictor. The probability density function of $y_{i}$ in Eq. (2) can be expressed

(10) $$P\left(Y_{i}=y_{i}|{\underset{˜}{x}}_{i}\right)=f\left(y_{i}|{\underset{˜}{x}}_{i}\right)=\frac{\theta {\left({\underset{˜}{x}}_{i}\right)}^{y_{i}}\exp\left(-\theta \left({\underset{˜}{x}}_{i}\right)\right)}{y_{i}!},\;\;y_{i}=0,\;1,\;2,\;\ldots$$

the mean value is

(11) $$E\left(Y_{i}|{\underset{˜}{x}}_{i}\right)=\theta \left({\underset{˜}{x}}_{i}\right)=\exp\left(m\left({\underset{˜}{x}}_{i}\right)\right)$$

Eq. (11) is substituted into Eq. (10) as follows

(12) $$f\left(y_{i}|{\underset{˜}{x}}_{i}\right)=\frac{{\left(m\left({\underset{˜}{x}}_{i}\right)\right)}^{y_{i}}\exp\left(-\exp m\left({\underset{˜}{x}}_{i}\right)\right)}{y_{i}!},\;\;y_{i}=0,\;1,\;2,\;\ldots$$

where $m({\underset{˜}{x}}_{i})$ is an unknown function that we aim to estimate using locally kernel weighted maximum likelihood estimator method. By applying for a Taylor expansion of degree one as an approximation for $m({\underset{˜}{x}}_{i})$, we have

(13) $$m\left({\underset{˜}{x}}_{i}\right)=\sum_{j=1}^{p}\sum_{i=1}^{n}\left\{\beta_{0j}\left(x_{0j}\right)+\beta_{1j}\left(x_{0j}\right)\left(x_{ij}-x_{0j}\right)\right\};x_{ij}\in \left(x_{0j}-h,x_{0j}+h\right)$$

$m\left(x_{1j}\right),m\left(x_{2j}\right),\cdots ,m\left(x_{nj}\right)$ is the local linear function in predictor variable x as many as p. Eq. (13) can be expressed in vector form, which is as follows:

(14) $$m\left({\underset{˜}{x}}_{i}\right)={\underset{˜}{x}}_{i}^{T}\underset{˜}{\beta }$$

With

$m\left({\underset{˜}{x}}_{i}\right)=\left[\begin{array}{c} m(x_{1j})\\ m(x_{2j})\\ \vdots \\ m(x_{nj}) \end{array}\right]$ ; ${\underset{˜}{x}}_{i}={\left[\begin{array}{c} {\underset{˜}{x}}_{1}\\ {\underset{˜}{x}}_{2}\\ \vdots \\ {\underset{˜}{x}}_{n} \end{array}\right]}^{T}={\left[\begin{array}{ccccc} 1 & \left(x_{11}-x_{01}\right) & \left(x_{12}-x_{02}\right) & \cdots & \left(x_{1p}-x_{0p}\right)\\ 1 & \left(x_{21}-x_{01}\right) & \left(x_{22}-x_{02}\right) & \cdots & \left(x_{2p}-x_{0p}\right)\\ \vdots & \vdots & \vdots & \ddots & \vdots \\ 1 & \left(x_{n1}-x_{01}\right) & \left(x_{n2}-x_{02}\right) & \cdots & \left(x_{np}-x_{0p}\right) \end{array}\right]}^{T}$;

$$\underset{˜}{\beta }={\left[\begin{array}{ccccccc} \beta_{01}\left(x_{01}\right) & \beta_{11}\left(x_{01}\right) & \beta_{02}\left(x_{02}\right) & \beta_{12}\left(x_{02}\right) & \cdots & \beta_{0p}\left(x_{0p}\right) & \beta_{1p}\left(x_{0p}\right) \end{array}\right]}^{T}$$

Therefore, Eq. (14) is substituted into Eq. (12),

(15) $$f\left(y_{i}|{\underset{˜}{x}}_{i}\right)=\frac{{\left({\underset{˜}{x}}_{i}^{T}\underset{˜}{\beta }\right)}^{y_{i}}\exp\left(-\exp\left({\underset{˜}{x}}_{i}^{T}\underset{˜}{\beta }\right)\right)}{y_{i}!},\;\;y_{i}=0,\;1,\;2,\;\ldots$$

Definition 1 *Suppose we are given an observation data for the response variable and predictor variable,* $x_{ij};i=1,2,\;\cdots ,n;\;j=1,2,\cdots ,p$ *, and* h>0 *are bandwidth, we have Kernel function is*

(16) $$f\left({\underset{˜}{x}}_{i}\right)=\prod_{j=1}^{p}K_{hj}(x_{ij}-x_{0j})$$

Let we have the Kernel function in Eq. (16) and the probability density function of $y_{i}$ in Eq. (10) then the local likelihood function can be written as

(17) $$\begin{array}{ccc} L(\underset{˜}{\beta },x_{0j}) & = & \prod_{i=1}^{n}{\left[f(y_{i}|{\underset{˜}{x}}_{i})\right]}^{\prod_{j=1}^{p}K_{hj}\left(x_{ij}-x_{0j}\right)}\\ & = & {\prod_{i=1}^{n}\left[\frac{\exp\left(y_{i}{\underset{˜}{x}}_{i}^{T}\underset{˜}{\beta }\right)-\exp\left({\underset{˜}{x}}_{i}^{T}\underset{˜}{\beta }\right)}{y_{i}}\right]}^{\prod_{j=1}^{p}K_{hj}\left(x_{ij}-x_{0j}\right)} \end{array}$$

Theorem 1 *The maximum likelihood estimator for the multi-predictor Poisson local linear model can be obtained by maximizing the* log*-likelihood function presented in* Eq. (18)*. By taking the natural logarithm of both sides of* Eq. (17)*, a local* log*-likelihood function is derived, which is given by*

(18) $$\ell \left(\underset{˜}{\beta },x_{0j}\right)=\prod_{j=1}^{p}\sum_{i=1}^{n}K_{h}\left(x_{ij}-x_{0j}\right)\left\{\left[\beta_{0j}\left(x_{0j}\right)+\beta_{1j}\left(x_{0j}\right)\left(x_{ij}-x_{0j}\right)\right]y_{i}-\exp\left[\beta_{0j}\left(x_{0j}\right)+\beta_{1j}\left(x_{0j}\right)\left(x_{ij}-x_{0j}\right)\right]-\ln\left(y_{i}!\right)\right\}$$

**Proof**. Based on (12), (16), we have the log local likelihood function

(19) $$\begin{array}{ccc} \ell (\underset{˜}{\beta },x_{0j}) & = & \ln l\left(\underset{˜}{\beta },x_{0j}\right)=\ln{\left(\prod_{i=1}^{n}f(y_{i}\left|{\underset{˜}{x}}_{i}\right)\right)}^{{}^{\prod_{j=1}^{p}K_{hj}\left(x_{ij}-x_{0j}\right)}}\\ & = & \ln{\left(\frac{\left[\exp\sum_{i=1}^{n}y_{i}\left({\underset{˜}{x}}_{i}^{T}\underset{˜}{\beta }\right)\right]\exp\left[-\sum_{i=1}^{n}\exp\left({\underset{˜}{x}}_{i}^{T}\underset{˜}{\beta }\right)\right]}{\prod_{i=1}^{n}y_{i}!}\right)}^{{}^{\prod_{j=1}^{p}K_{hj}\left(x_{ij}-x_{0j}\right)}}\\ & = & \prod_{j=1}^{p}K_{hj}\left(x_{ij}-x_{0j}\right)\left(\frac{\sum_{i=1}^{n}y_{i}\left({\underset{˜}{x}}_{i}^{T}\underset{˜}{\beta }\right)-\sum_{i=1}^{n}\exp\left({\underset{˜}{x}}_{i}^{T}\underset{˜}{\beta }\right)}{\sum_{i=1}^{n}\ln y_{i}!}\right)\\ & = & \prod_{j=1}^{p}\sum_{i=1}^{n}K_{hj}\left(x_{ij}-x_{0j}\right)\left(\left({\underset{˜}{x}}_{i}^{T}\underset{˜}{\beta }\right)y_{i}-\exp\left({\underset{˜}{x}}_{i}^{T}\underset{˜}{\beta }\right)-\ln y_{i}!\right) \end{array}$$

Eq. (13) substituted into Eq. (19), we obtained

(20) $$\ell \left(\underset{˜}{\beta },x_{0j}\right)=\prod_{j=1}^{p}\sum_{i=1}^{n}K_{h}\left(x_{ij}-x_{0j}\right)\left\{\left[\beta_{0j}\left(x_{0j}\right)+\beta_{1j}\left(x_{0j}\right)\left(x_{ij}-x_{0j}\right)\right]y_{i}-\exp\left[\beta_{0j}\left(x_{0j}\right)+\beta_{1j}\left(x_{0j}\right)\left(x_{ij}-x_{0j}\right)\right]-\ln\left(y_{i}!\right)\right\}$$

The estimation of the parameters in the multi-predictor Poisson regression model can be obtained using the locally kernel weighted maximum likelihood estimator method. Lemma 1 can be applied in Theorem 1 to obtain the results for estimating the parameters $\underset{˜}{\beta }$​.

Theorem 2 *For paired pair observational data*$({\underset{˜}{x}}_{i},y_{i}),\;i=1,\;2,\;\cdots ,\;n$*and*${\underset{˜}{x}}_{i}=\left[\begin{array}{cccc} x_{i1} & x_{i2} & x_{i3} & x_{ip} \end{array}\right]$*,*$y_{i}$*is the count response that follows the Poisson distribution and the multi-predictor Poisson regression model as given in*Eq. (14)*and the local* log *likelihood function as in* Eq. (20)*, estimator for the parameters* $\underset{˜}{\beta }$ *can be obtained from the solution of the following equation*

$$\frac{\partial \ell (\underset{˜}{\beta },x_{0j})}{\partial \beta_{0j}\left(x_{0j}\right)}=0$$

(21) $$\prod_{j=1}^{p}\sum_{i=1}^{n}K_{hj}\left(x_{ij}-x_{0j}\right)\left\{y_{i}-\exp\left[\beta_{0j}\left(x_{0j}\right)+\beta_{1j}\left(x_{0j}\right)\left(x_{ij}-x_{0j}\right)\right]\right\}=0$$

The first derivatives of the local log likelihood function in Eq. (20) with respect $\beta_{1j}\left(x_{0j}\right)$ is

$$\frac{\partial L(\underset{˜}{\beta },x_{0j})}{\partial \beta_{1j}\left(x_{0j}\right)}=0$$

(22) $$\prod_{j=1}^{p}\sum_{i=1}^{n}K_{hj}\left(x_{ij}-x_{0j}\right)\left\{y_{i}\left(x_{ij}-x_{0j}\right)-\left(x_{ij}-x_{0j}\right)\exp\left[\beta_{0j}\left(x_{0j}\right)+\beta_{1j}\left(x_{0j}\right)\left(x_{ij}-x_{0j}\right)\right]\right\}=0$$

Eq. (21) and [22] represents implicit function. Thus, an iterative process is required to obtain their solutions. An alternative approach to solve the likelihood equation to find the maximum likelihood estimator is to apply the Newton-Raphson iterative method [41].

Definition 2 *Let the first derivatives of the local* log *likelihood function with respect to the parameter* $\underset{˜}{\beta }$ *(as shown in* Eq. (21) *and (*22*),* $x_{ij};\;\;j=1,2,...,p$ *is predictor variable, the gradient vector is*

(23) $$\begin{array}{ccc} g(\underset{˜}{\beta }) & = & \frac{\partial \ell (\underset{˜}{\beta },x_{0j})}{\partial (\underset{˜}{\beta })}\\ & = & {\left[\begin{array}{ccccc} \frac{\partial \ell (\underset{˜}{\beta },x_{0j})}{\partial \beta_{0j}\left(x_{0j}\right)} & \frac{\partial \ell (\underset{˜}{\beta },x_{01})}{\partial \beta_{11}\left(x_{01}\right)} & \frac{\partial \ell (\underset{˜}{\beta },x_{02})}{\partial \beta_{12}\left(x_{02}\right)} & \cdots & \frac{\partial \ell (\underset{˜}{\beta },x_{0p})}{\partial \beta_{1p}\left(x_{0p}\right)} \end{array}\right]}^{T}\\ & = & \prod_{j=1}^{p}\sum_{i=1}^{n}\left(x_{ij}-x_{0j}\right)K_{hj}\left(x_{ij}-x_{0j}\right)\left(y_{i}-\exp\left[{\underset{˜}{x}}_{i}^{T}\beta \right]\right) \end{array}$$

Definition 3 *Let the first derivatives of the local* log *likelihood function with respect to the parameter* $\underset{˜}{\beta }$ *(as shown in* Eq. (21) *and (*22*)), the Hessian matrix is*

(24) $$H\left(\underset{˜}{\beta }\right)=\left[\begin{array}{cccc} \frac{\partial^{2}\ell \left(\underset{˜}{\beta },x_{0j}\right)}{\partial \beta_{01}^{2}\left(x_{0j}\right)} & \frac{\partial^{2}\ell \left(\underset{˜}{\beta },x_{0j}\right)}{\partial \beta_{01}\partial \beta_{11}\left(x_{0j}\right)} & \cdots & \frac{\partial^{2}\ell \left(\underset{˜}{\beta },x_{0j}\right)}{\partial \beta_{01}\partial \beta_{1p}}\\ \frac{\partial^{2}\ell \left(\underset{˜}{\beta },x_{0j}\right)}{\partial \beta_{11}\partial \beta_{01}\left(x_{0j}\right)} & \frac{\partial^{2}\ell \left(\underset{˜}{\beta },x_{0j}\right)}{\partial \beta_{11}^{2}\left(x_{0j}\right)} & \cdots & \frac{\partial^{2}\ell \left(\underset{˜}{\beta },x_{0j}\right)}{\partial \beta_{11}\partial \beta_{1p}\left(x_{0j}\right)}\\ \vdots & \vdots & \ddots & \vdots \\ \frac{\partial^{2}\ell \left(\underset{˜}{\beta },x_{0j}\right)}{\partial \beta_{1p}\partial \beta_{0p}\left(x_{0j}\right)} & \frac{\partial^{2}\ell \left(\underset{˜}{\beta },x_{0j}\right)}{\partial \beta_{1p}\partial \beta_{11}\left(x_{0j}\right)} & \cdots & \frac{\partial^{2}\ell \left(\underset{˜}{\beta },x_{0j}\right)}{\partial \beta_{1p}^{2}\left(x_{0j}\right)} \end{array}\right]$$

The elements of the Hessian matrix in Eq. (24) can be expressed in a general form, namely

(25) $$\frac{\partial^{2}\ell \left(\underset{˜}{\beta },x_{0j}\right)}{\partial {\underset{˜}{\beta }}^{2}\left(x_{0j}\right)}=-\prod_{j=1}^{p}\sum_{i=1}^{n}K_{hj}\left(x_{ij}-x_{0j}\right){\left(x_{ij}-x_{0j}\right)}^{2}\exp\left({\underset{˜}{x}}_{i}^{T}\underset{˜}{\beta }\right)$$

(26) $$\frac{\partial^{2}\ell \left(\underset{˜}{\beta },x_{0j}\right)}{\partial \beta_{0j}\partial \beta_{1k}}=-\prod_{j=1}^{p}\sum_{i=1}^{n}\left(x_{ij}-x_{0j}\right)\left(x_{ik}-x_{0j}\right)K_{hj}\left(x_{ij}-x_{0j}\right)\exp\left({\underset{˜}{x}}_{i}^{T}\underset{˜}{\beta }\right),j,k=0,1,2,...,p;j\neq k$$

Based on Eq. (25) and (26), the Hessian matrix can be expressed as follows

(27) $$H\left(\underset{˜}{\beta }\right)=-X^{T}\mathrm{VX}$$

*where*

(28) $$X=\left[\begin{array}{ccccc} 1 & \left(x_{11}-x_{01}\right) & \left(x_{12}-x_{02}\right) & \cdots & \left(x_{1p}-x_{0p}\right)\\ 1 & \left(x_{21}-x_{01}\right) & \left(x_{22}-x_{02}\right) & \cdots & \left(x_{2p}-x_{0p}\right)\\ \vdots & \vdots & \vdots & \ddots & \vdots \\ 1 & \left(x_{n1}-x_{01}\right) & \left(x_{n2}-x_{02}\right) & \cdots & \left(x_{np}-x_{0p}\right) \end{array}\right]$$

*and*

(29) $$V=\left[\begin{array}{cccc} \exp({{\underset{˜}{x}}_{1}}^{T}\underset{˜}{\beta }) & 0 & \cdots & 0\\ 0 & \exp({{\underset{˜}{x}}_{2}}^{T}\underset{˜}{\beta }) & \cdots & 0\\ \vdots & \vdots & \ddots & \vdots \\ 0 & 0 & 0 & \exp({{\underset{˜}{x}}_{n}}^{T}\underset{˜}{\beta }) \end{array}\right]$$

Fisher Information matrix can be defined as

(30) $$I\left(\hat{\underset{˜}{\beta }}\right)=-E\left(H(\hat{\underset{˜}{\beta }}\right))=-H(\hat{\underset{˜}{\beta }})$$

Based on Eq. (27), Eq. (30) can be expressed as

(31) $$I(\hat{\underset{˜}{\beta }})=X^{T}\hat{V}X$$

Based on the elements of the gradient vector and the Hessian matrix obtained from Eq. (24) and (25), the Newton-Raphson iteration can be performed to obtain the parameter estimator using the algorithm

(32) $${\hat{\underset{˜}{\beta }}}^{\left(m+1\right)}={\hat{\underset{˜}{\beta }}}^{\left(m\right)}-{\left[H({\hat{\underset{˜}{\beta }}}^{\left(m\right)})\right]}^{-1}g({\hat{\underset{˜}{\beta }}}^{\left(m\right)});m=0,1,2,..$$

The iteration process begins by determining the initial estimated value of the parameter

(33) $${\hat{\underset{˜}{\beta }}}^{\left(0\right)}\left(x_{0j}\right)={\left[\begin{array}{cccc} {\hat{\underset{˜}{\beta }}}_{0j}^{\left(0\right)}\left(x_{0j}\right) & {\hat{\underset{˜}{\beta }}}_{11}^{\left(0\right)}\left(x_{0j}\right) & \cdots & {\hat{\underset{˜}{\beta }}}_{1p}^{\left(0\right)}\left(x_{0j}\right) \end{array}\right]}^{T}$$

The iteration is stopped at the ${\left(m+1\right)}^{-th}$ iteration, which is $∥{\hat{\underset{˜}{\beta }}}^{\left(m+1\right)}-{\hat{\underset{˜}{\beta }}}^{\left(m\right)}∥\leq \varepsilon$, where ε is a small positive number. The initial estimate of the parameter is calculated using Ordinary Least Squares (OLS),

(34) $${\hat{\underset{˜}{\beta }}}^{\left(0\right)}={\left(X^{*T}X^{*}\right)}^{-1}X^{*T}{\underset{˜}{Y}}^{*}$$

where

$$X^{*}=\left[\begin{array}{ccccc} 1 & x_{11} & x_{12} & \cdots & x_{1p}\\ 1 & x_{21} & x_{22} & \cdots & x_{2p}\\ \vdots & \vdots & \vdots & \ddots & \vdots \\ 1 & x_{n1} & x_{n2} & \cdots & x_{np} \end{array}\right]\;and\;\underset{˜}{Y}={\left[\begin{array}{cccc} y_{1} & y_{2} & \cdots & y_{n} \end{array}\right]}^{T}.$$

Definition 4 *The optimal bandwidth selection is based on the highest value of the maximum likelihood cross validation (MLCV).*

(35) $$MLCV\left(h\right)=\sum_{i=1}^{n}\{{\hat{m}}_{-1}\left({\underset{˜}{x}}_{i}\right)y_{i}-\exp({\hat{m}}_{-1}({\underset{˜}{x}}_{i}))-\ln y_{i}!\}$$

where ${\hat{m}}_{-i}\left({\underset{˜}{x}}_{i}\right)$ is the estimated function at the point $x_{i}$ without including the i^th^ data.

### Application for stunting cases in east kalimantan

The study data was secondary data collected from the Health Office of West Kutai, Paser, and Berau Regencies East Kalimantan Province in 2023. The research sample size was 39 districts. The data consists of the observation result data of the response variable and predictor. The response variable was the stunting cases (Y), and the predictor involves percentage of infants receiving exclusive breastfeeding (X_1_), and the percentage of children receiving complete basic immunization (X_2_). The data analysis technique used was multi-predictor Poisson regression modeling using a local linear approach, and computation using Open-Source Software (OSS) R. The data description is presented through the descriptive statistics shown in Table 1.

**Table 1:** Descriptive Statistics of the Study Data.

Variable	Average	Minimum	Maximum	Standar Deviation
The stunting cases (Y)	110	8	361	96.7
Percentage of infants receiving exlusive breastfeeding $\left(X_{1}\right)$	4.06	0	21.43	4.70
Persentage of children receiving complete basic immunization $\left(X_{2}\right)$	7.28	0.75	30.01	6.15

According to Table 1, the stunting cases in East Kalimantan Province is relatively small, namely 110 children, the stunting cases can be considered to follow a Poisson distribution.

**Fig. 1:**
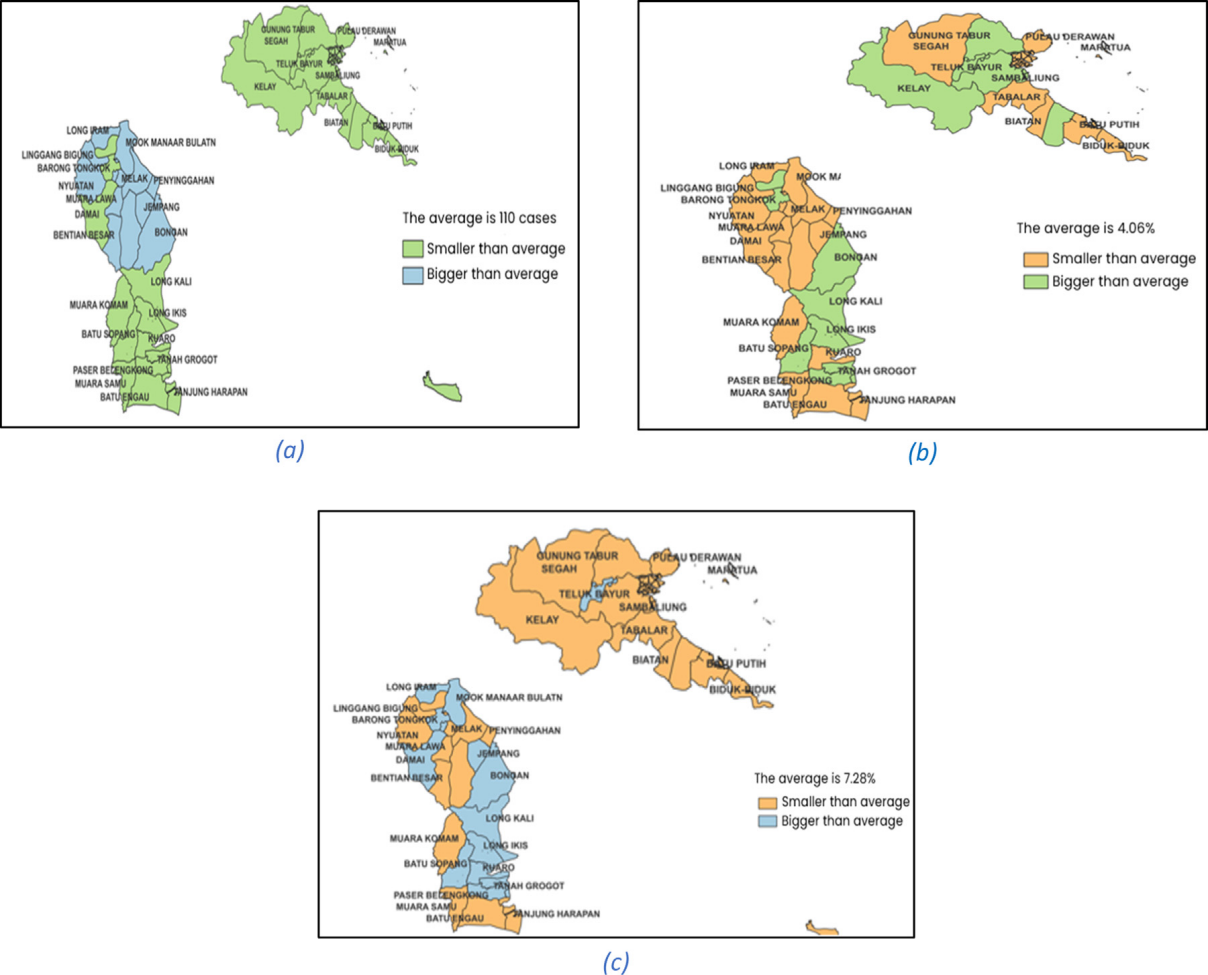
The distribution map of the stunting cases (a), percentage of infants receiving exlusive breastfeeding (b) percentage of children receiving complete basic immunization (c) in East Kalimantan Province 2023.

The distribution of the stunting cases, percentage of infants receiving exclusive breastfeeding, and percentage of children receiving complete basic immunization in East Kalimantan Province 2023 are presented in Fig. 1. The classification of stunting cases is divided into two categories: those that are below or above the average number of cases in the province. Additionally, the Poisson regression model for stunting cases can involve two predictors as shown in Table 1. The parameter estimation method used is MLE, and the maximum likelihood estimator is determined using the iterative Newton-Raphson algorithm. The Poisson regression, known as global model, which represents the mean fit model of the stunting cases in East Kalimantan 2023, can be expressed as follows:

(36) $$m\left(x_{i}\right)=\exp\left(4.625468-0.0000399x_{i1}-0.084710x_{i2}\right);i=1,2,...,\;39$$

To obtain the better model, the multi-predictor Poisson regression model based on local linear estimator is proposed. Multi-predictor Poisson regression model based on local linear estimator for two predictor variables can be expressed in Eq. (37).

(37) $$\begin{array}{c} m\left({\underset{∼}{x}}_{i}\right)=\beta_{01}\left(x_{01}\right)+\beta_{i1}\left(x_{01}\right)\left(x_{i1}-x_{01}\right)+\beta_{02}\left(x_{02}\right)+\beta_{i2}\left(x_{02}\right)\left(x_{i2}-x_{02}\right),i=1,2,...,\;39 \end{array}$$

Parameter estimation is conducted at every observation point using weighted Kernel function, and the ML estimator is found numerically using iterative Newton-Raphson method. The parameter estimation process begins by calculating the weighted kernel function, determining the optimal bandwidth, and subsequently employing the iterative Newton-Raphson algorithm. The multi-predictor Poisson regression model based on local linear estimator result at each observation point is the local model called local linear multi-predictor Poisson regression model which is stated the stunting cases average model at a regency in East Kalimantan 2023. In the local linear estimator, we used MLCV for selection optimal bandwidth (h) as indicator the best model. These results are presented in Table 2.

**Table 2:** Bandwidth and MLCV value.

*X_1_*		*X_2_*	
Bandwidth	MLCV	Bandwidth	MLCV
0.55	3.126820	0.34	0.360435
**0.56**	**5.181427**	**0.35**	**2.202866**
0.57	5.090332	0.36	2.064573

Based on optimal bandwidth, parameter estimation of local linear multi-predictor Poisson regression model is conducted. Result of maximum likelihood estimator for every observation point, namely district in regency of West Kutai, Paser, and Berau in East Kalimantan 2023 displayed in Table 3.

**Table 3:** ML Estimator of local linear multi-predictor Poisson regression model.

Regency	i	District	Parameter		
			$\beta_{0i}$	$\beta_{1i}$	$\beta_{2i}$
West Kutai	1	Bongan	4.537467	−0.000047	−0.006071
	2	Linggang Bigung	4.525378	−0.000035	−0.007083
	3	Penyinggahan	4.538571	−0.000142	−0.006054
	4	Muara Pahu	4.528465	−0.000021	−0.006083
	5	Tering Seberang	3.547468	−0.000038	−0.006088
	6	Muara Lawa	3.436451	−0.000081	−0.005147
	7	Damai	3.636485	−0.000047	−0.005075
	8	Barong Tongkok	4.477469	−0.000349	−0.008562
	9	Long Iram	4.428541	−0.000042	−0.005035
	10	Melak	4.537467	−0.000021	−0.005179
	11	Siluq Ngurai	3.538653	−0.000045	−0.006071
	12	Nyuatan	4.527486	−0.000042	−0.005043
	13	Jempang	4.537452	−0.000039	−0.006071
	14	Sekolaq Darat	4.437348	−0.000047	−0.006045
	15	Mook Manaar Bulatn	3.537467	−0.000036	−0.006056
	16	Bentian Besar	3.537467	−0.000053	−0.006084

Paser	17	Batu Sompang	3.517467	−0.000039	−0.006034
	18	Muara Samu	3.417467	−0.000063	−0.006053
	19	Batu Engau	4.537467	−0.000057	−0.006053
	20	Tanjung Harapan	3.937467	−0.000049	−0.007072
	21	Paser Belengkong	4.998712	−0.000081	−0.006092
	22	Tanah Grogot	4.998887	−0.000052	−0.006077
	23	Kuaro	4.998871	−0.000034	−0.006072
	24	Long Ikis	4.988469	−0.000062	−0.006063
	25	Muara Komam	4.998467	−0.000081	−0.006081
	26	Long Kali	4.938466	−0.000057	−0.005173

Berau	27	Tanjung Redep	4.987887	−0.000074	−0.005091
	28	Teluk Bayur	4.998467	−0.000039	−0.006077
	29	Sambaliung	4.792747	−0.000073	−0.006071
	30	Gunung Tabur	4.998879	−0.000048	−0.006073
	31	Pulau Derawan	4.537467	−0.000055	−0.006081
	32	Maratu	3.537467	−0.000047	−0.006046
	33	Talisayan	4.923746	−0.000231	−0.006084
	34	Biatan Lempake	4.335034	−0.000046	−0.006086
	35	Tabalar	4.537467	−0.000041	−0.006072
	36	Batu Putih	3.637467	−0.000038	−0.006071
	37	Biduk-Biduk	4.537467	−0.000024	−0.006062
	38	Kelay	3.537467	−0.000028	−0.005076
	39	Segah	4.941467	−0.000032	−0.008072

Based on Table 3, and referring Eq. (33), it is obtained the stunting cases average model at every discrict in East Kalimantan. For example, the stunting cases average model at Discrict of Bongan (1-th observation), Discrict of Long Kali (26-th observation), and Discrict of Segah (39-th observation), respectively is :

$$m\left({\underset{˜}{x}}_{1}\right)=\exp(4.537467-0.000047\left(x_{11}-5.47945\right)-0.006071\left(x_{12}-12.8564\right);$$

(38) $$0.298025<x_{11}<10.660879;10.653586<x_{12}<15.059318$$

$$m\left({\underset{˜}{x}}_{26}\right)=\exp(4.938466-0.000057\left(x_{26.1}-5.628198\right)-0.005173\left(x_{26.2}-7.950311\right);$$

(39) $$0.446771<x_{26.1}<10.809625;5.747445<x_{26.2}<10.153177$$

and

$$m\left({\underset{˜}{x}}_{39}\right)=\exp(4.941467-0.000032\left(x_{27.1}-1.838565\right)-0.008072\left(x_{27.2}-6.680541\right);$$

(40) $$0<x_{39.1}<7.019992;4.477675<x_{39.2}<8.883407$$

**Fig. 2:**
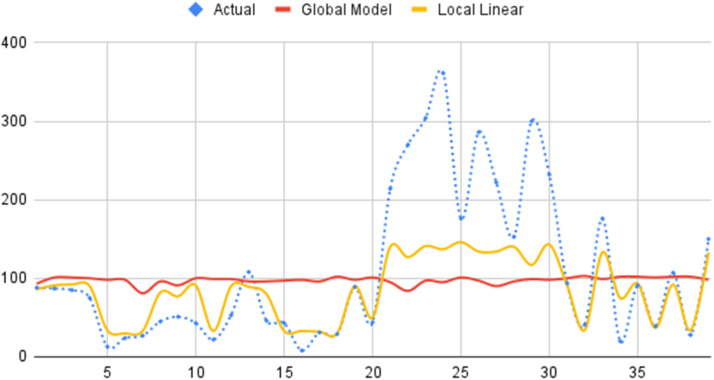
Estimation of stunting cases in East Kalimantan 2023 using global model and local linear multi-predictor Poisson regression model.

Based on Eq (38), if percentage of infants receiving exclusive breastfeeding (X_1_) is 6.47945 and the percentage of children receiving complete basic immunization (X_2_) is 13.8564 then the stunting cases average in District of Bongan is 93. Performance multi-predictor Poisson regression based on local linear estimator can be shown in Fig. 2.

Fig. 2 reveals highly fluctuating actual data, represented by the blue line. Measured values exhibit significant variations between observations, with peaks and troughs. The global model (red line) generally captures the overall trend but struggles to accurately reflect these fluctuations. This model's predictions tend to be relatively constant. In contrast, the local linear model (yellow line) demonstrates superior performance in capturing data fluctuations, effectively tracking local trends. The global model's MLCV value was 9489.160018, while the local linear model's MLCV was 1811.482859.

### Conclusion

Based on this study, we developed a multi-predictor Poisson regression model using a local linear approach. This method offers several advantages, such as the ability to estimate the function at each point, resulting in estimates that closely align with the observed data patterns. It also adheres to the principle of parsimony, making it easier to interpret and less reliant on large datasets. We detailed the procedure for obtaining the regression curve estimates at a given point, where the locally kernel-weighted maximum likelihood estimator is applied, and parameter estimates are computed using the Newton-Raphson iteration method. We apply the regression model that we propose to Health data, the response variable was the stunting cases (Y), and the predictor involves the percentage of infants receiving exclusive breastfeeding (X_1_), and the percentage of children receiving complete basic immunization (X_2_). The model evaluation results show the superiority of local linear models in handling data with a high level of variability. The ability of this model to capture local fluctuations has significant implications in modeling stunting problems. Thus, the use of local linear models can increase prediction accuracy and understanding of the phenomenon being studied. In future work, we plan to extend the model to incorporate polynomials, such as in a multi-predictor Poisson regression model using a polynomial local approach.

## Limitations

Not applicable.

## Ethics statements

This research, titled ``Locally Kernel Weighted Maximum Likelihood Estimator for Local Linear Multi-Predictor Poisson Regression'', did not involve any human participants, animals, or personal data, and thus did not require formal ethical approval. The study focuses on the development and application of statistical methodologies, specifically designed to enhance Poisson regression models based on local linear approach. The research adheres to the highest standards of academic integrity and transparency, with no conflicts of interest, and aims to contribute to the broader scientific understanding of advanced statistical techniques.

## Credit Author Statement

**Darnah:** Conceptualization, Methodology, Validity test, Writing-Preparation of the first draft, and Supervision. **Memi Nor Hayati:** Methodology, Validity test. **Sri Wahyuningsih:** Methodology, Validity test. **Iriyani Kamaruddin:** Methodology, Validity test. **Suyitno:** Conceptualization, Methodology, Validity test. **Andrea Tri Rian Dani:** Writing-Review and Editing. **Rito Goejantoro:** Methodology, Validity test. **Desi Yuniarti:** Writing-Review and Editing. **Fidia Deny Tisna Amijaya:** Methodology and Validity test. **Ika Purnamasari:** Conceptualization and Methodology. **Meiliyani Siringoringo:** Writing-Review and Editing. **Surya Prangga:** Conceptualization and Validity test. **Ratna Kusuma:** Supervision and Funding Acquisition. **Rahmawati Munir:** Validity test and Project Administration.

## Declaration of Competing Interest

The authors declare that they have no known competing financial interests or personal relationships that could have appeared to influence the work reported in this paper.

## Data Availability

The data that has been used is confidential.
